# Comparing the Potential of Multispectral and Hyperspectral Data for Monitoring Oil Spill Impact

**DOI:** 10.3390/s18020558

**Published:** 2018-02-12

**Authors:** Shruti Khanna, Maria J. Santos, Susan L. Ustin, Kristen Shapiro, Paul J. Haverkamp, Mui Lay

**Affiliations:** 1 Center for Spatial Technologies and Remote Sensing, Department of Land Air and Water Resources, University of California, One Shields Avenue, Davis, CA 95616, USA; slustin@ucdavis.edu (S.L.U.); kdshapiro@ucdavis.edu (K.S.); pjhav@ucdavis.edu (P.J.H.); mclay@ucdavis.edu (M.L.); 2 Department of Innovation, Environmental and Energy Sciences, Utrecht University, 3584 CS Utrecht, The Netherlands; maria.dossantos@geo.uzh.ch; 3 Department of Geography, University of Zürich, 8057 Zürich, Switzerland; 4 Department of Evolutionary Biology and Environmental Studies, University of Zürich, 8057 Zürich, Switzerland

**Keywords:** vegetation indices, LANDSAT, WorldView-2, RapidEye, AVIRIS

## Abstract

Oil spills from offshore drilling and coastal refineries often cause significant degradation of coastal environments. Early oil detection may prevent losses and speed up recovery if monitoring of the initial oil extent, oil impact, and recovery are in place. Satellite imagery data can provide a cost-effective alternative to expensive airborne imagery or labor intensive field campaigns for monitoring effects of oil spills on wetlands. However, these satellite data may be restricted in their ability to detect and map ecosystem recovery post-spill given their spectral measurement properties and temporal frequency. In this study, we assessed whether spatial and spectral resolution, and other sensor characteristics influence the ability to detect and map vegetation stress and mortality due to oil. We compared how well three satellite multispectral sensors: WorldView2, RapidEye and Landsat EMT+, match the ability of the airborne hyperspectral AVIRIS sensor to map oil-induced vegetation stress, recovery, and mortality after the DeepWater Horizon oil spill in the Gulf of Mexico in 2010. We found that finer spatial resolution (3.5 m) provided better delineation of the oil-impacted wetlands and better detection of vegetation stress along oiled shorelines in saltmarsh wetland ecosystems. As spatial resolution become coarser (3.5 m to 30 m) the ability to accurately detect and map stressed vegetation decreased. Spectral resolution did improve the detection and mapping of oil-impacted wetlands but less strongly than spatial resolution, suggesting that broad-band data may be sufficient to detect and map oil-impacted wetlands. AVIRIS narrow-band data performs better detecting vegetation stress, followed by WorldView2, RapidEye and then Landsat 15 m (pan sharpened) data. Higher quality sensor optics and higher signal-to-noise ratio (SNR) may also improve detection and mapping of oil-impacted wetlands; we found that resampled coarser resolution AVIRIS data with higher SNR performed better than either of the three satellite sensors. The ability to acquire imagery during certain times (midday, low tide, etc.) or a certain date (cloud-free, etc.) is also important in these tidal wetlands; WorldView2 imagery captured at high-tide detected a narrower band of shoreline affected by oil likely because some of the impacted wetland was below the tideline. These results suggest that while multispectral data may be sufficient for detecting the extent of oil-impacted wetlands, high spectral and spatial resolution, high-quality sensor characteristics, and the ability to control time of image acquisition may improve assessment and monitoring of vegetation stress and recovery post oil spills.

## 1. Introduction

Coastal wetlands are important but vulnerable ecosystems that provide valuable ecosystem services such as protection from storms and storm surges, support for fisheries and seafood industries, nursery sites for juvenile fish, carbon storage, nutrient cycling, waterfowl habitat and other key services [1,2,3,4,5,6,7,8]. Coastal wetlands are also subject to relatively frequent disturbances such as hurricanes and other storms, changes in sea level, and contamination from runoff, tidal inputs, and oil spills, all of which cause vegetation stress and wetland degradation [9,10,11,12,13]. This can cause lasting damage for years to decades [13,14,15,16]. Since 1973, both the volume and number of oil spill incidents has declined in the United States [17]. However, large oil spill events still cause disproportionate impacts in the wetlands where they occur. The DeepWater Horizon (DWH) oil spill in the Gulf of Mexico in 2010 was the biggest oil spill in the US history and the second-largest in the world [18]. The DWH spill occurred in deep oceanic pipelines, which were difficult to contain. In total, 206 million gallons of oil were released over 85 days from 20 April to 19 July, which contaminated 572 miles of gulf shoreline [18]. The effects of the DWH oil spill on the Gulf of Mexico wetlands are likely still ongoing, as demonstrated by several recent studies [16,19,20,21,22,23,24].

Oil is highly detrimental to plant health, both directly and indirectly. Oil affects plant metabolism directly as coating the leaves prevents gas exchange between the leaves and the atmosphere, significantly increasing leaf and plant mortality [25,26,27,28,29]. Oil also affects plants indirectly when present in the soil by reducing oxygen exchange between the atmosphere and soil, affecting the microbial community and nutrient cycling [15,25,26,30]. At the ecosystem level, post-oil spill senescent and dead plant material slowly erodes resulting in a transition from wetland to bare soil or water [25]. The recovery from oil effects can take months to several years [13,15,31], especially since different wetland communities differ in their susceptibility to oil impacts [14,32,33].

As plants become increasingly stressed, they lose pigments, water, and ultimately leaves, all of which can be measured through changes in their reflectance [34,35,36,37,38]. Plant stress affects plant reflectance in regions of the electromagnetic spectrum related to plant pigment concentration [35,36,37,39,40], water content [41,42,43], and leaf area or canopy density [44]. Most multispectral satellite sensors have at least four fundamental bands: Blue, Green, Red, and Near InfraRed (NIR) [45]. Vegetation indices based on these bands are effective in tracking both pigment and leaf area [46,47]. The Shortwave InfraRed (SWIR) band is available on some freely available multispectral sensors like Landsat or the European Space Agency’s Sentinel-2 satellites and is used to measure plant water content [43]. Vegetation indices calculated from imaging spectrometers can track all of these changes as demonstrated by Khanna et al. [20] after the DWH oil spill. Change in land cover due to loss of wetland vegetation can be tracked through “angle indices”, which are relatively insensitive to the confounding influence of soil moisture [44,48]. Thus, imaging spectrometer data is useful in mapping and monitoring oil spill impacts on wetland extent and oil induced vegetation stress [49,50,51,52]. 

Few studies have assessed the post-oil spill stress on wetland vegetation, and then, most have used imaging spectrometer data [20,49,53,54,55]. The objective of this study is to explore the potential for using easily available multispectral sensor data to detect and map vegetation stress and mortality due to oil contamination. We sought to determine whether oil-induced plant stress can be detected and monitored using multispectral satellite imagery with fine (WorldView-2 and RapidEye; 2 m and 5 m) and moderate spatial resolutions (Landsat ETM+; pan sharpened 15 m or 30 m). We contrast multispectral sensors with airborne AVIRIS imagery in their abilities to detect vegetation stress in oiled versus oil-free shores. Each sensor has different characteristics regarding spectral, spatial, and radiometric resolution, and sensor engineering, all of which might influence the potential of the sensor data to monitor oil spill impacts.

## 2. Data and Methods

### 2.1. Study Area

**Figure 1:**
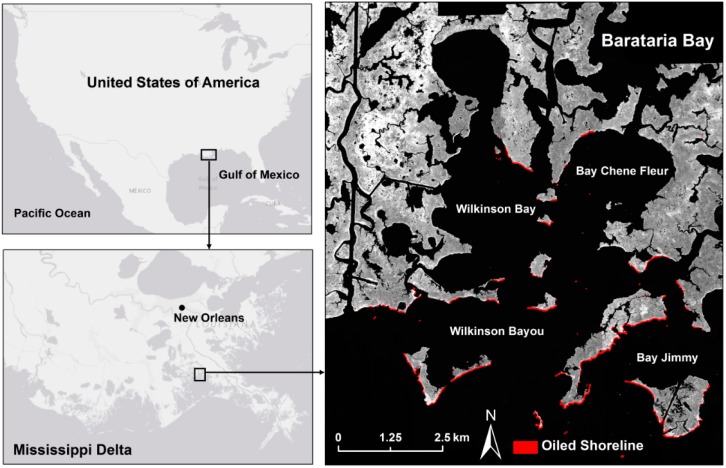
AVIRIS gray scale image of Barataria Bay and its location in the Mississippi Delta. Oil mapped using AVIRIS 2010 imagery is overlaid in red.

Barataria Bay is located approximately 160 km from the DWH oil spill site in an interlobe basin between the current Bird’s Foot delta and the abandoned Lafourche delta lobes [56]. The dominant plant species in the low intertidal saltmarshes is *Spartina alterniflora* (saltmarsh cordgrass) and *Juncus roemerianus* (needlegrass rush), with subdominants *Spartina patens* (salt meadow cordgrass), *Distichlis spicata* (saltgrass) and *Batis maritima* (saltwort) more common in the higher marsh [57]. As the DWH oil spill occurred offshore, the oil came in with the tide and primarily contaminated the seaward edges of the wetlands (Figure 1). Several studies mapped the oil spill extent in the ocean [58,59,60,61] and the wetlands [62], and its detrimental effects on the wetland vegetation [20,22].

### 2.2. Image Data and Preprocessing

AVIRIS data was acquired over Barataria Bay and used to map the presence of oil along the wetland shoreline and to detect plant stress due to oil contamination on vegetation [20]. Four flight-lines covering an area of 175 km^2^ (Table 1) were georeferenced by NASA Jet Propulsion Laboratory using information derived from inertial navigation data and GPS. Unfortunately, images georeferenced based on this information often suffer from residual misalignment by a few pixels or more, and this misalignment translated into displacements of the order of dozens of meters, whereas oil penetration and impact was unequal near the shore exhibiting sharp gradients on the scale of just a few meters [20,63]. Hence the 2010 images were further georectified to 1 m National Agricultural Imagery Program (NAIP) color infrared images collected in 2010. Images were also atmospherically calibrated using the Atmospheric CORrection Now algorithm (ACORN) 6, mode 1.5 (ImSpec LLC, Seattle, WA, USA) to apparent surface reflectance.

Seventeen multispectral WorldView2 images (Table 1) covering the same area as AVIRIS were atmospherically calibrated to apparent surface reflectance and mosaicked. Two RapidEye images were also calibrated to apparent surface reflectance and mosaicked. Finally, one Landsat ETM+ image was calibrated to apparent surface reflectance using ACORN 6, mode 5. Since both WorldView2 and RapidEye data were acquired off-nadir, we could not use ACORN for atmospheric correction. Hence we used Atmospheric Correction and Haze Reduction (ATCOR) [69] which allows input of non-nadir view angles. Moreover, it performs at least as well as ACORN and the results of the two calibration techniques are comparable, especially over a flat terrain such as Barataria Bay [70].

**Figure 2:**
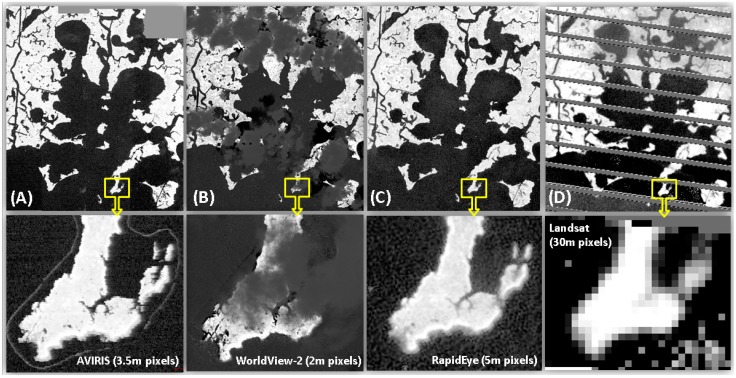
Barataria Bay gray scale image subset for all four sensors and a close view of a smaller region to highlight differences in image quality and spatial resolution (**A**) AVIRIS 3.5 m imagery, (**B**) WorldView2 2 m imagery, (**C**) RapidEye 5 m imagery, and (**D**) Landsat ETM+ 30 m imagery.

All images from the three multispectral sensors were subset to the spatial extent of the oil-affected section of the study area (Figure 2, 86 km^2^). Both WorldView2 and RapidEye image mosaics were further co-registered to the AVIRIS 2010 imagery using an automated image registration technique [71,72] to enable comparisons between the different sensors. The advantage of this technique is that areas of spurious change can be excluded, e.g., clouds in one image or shoreline changes with varying tidal stage. We were able to exclude these areas from calculations of displacement between images, hence the tidal stages of different images did not affect the accuracy of co-registration.

We mapped oil on exposed soil surfaces and dry vegetation, and water and land pixels in the AVIRIS 2010 images using a binary decision tree following methods in Khanna et al. [73]. Inputs to the decision tree included vegetation and angle indices and continuum removals [74] over two well-known oil absorption features centered at 2100 nm and 2300 nm [62]. Binary classes, land and water, were mapped in addition to oiled pixels.

The United States Geological Survey (USGS) conducted field data surveys in Barataria Bay on 10 July 2010 and again on 12–13 August 2010 [63]. At each 2 × 2 m survey point, they collected information on the vegetation species composition, canopy condition, presence of oil, and penetration of oil into the marsh. We randomly chose half the points for training the classifier to recognize oiled pixels and the other half to test the accuracy of detecting oiled pixels using Kappa statistics, and overall classification accuracy [75,76]. The results of this classification were first presented in Khanna et al. [20] showing that the oiled pixels were classified with an overall accuracy of 95% and a Kappa of 0.88.

### 2.3. Methods

#### 2.3.1. AVIRIS Hyperspectral Data

To compare the effect of sensor spectral and spatial resolution on detecting plant stress, we spectrally resampled AVIRIS hyperspectral data to simulate the WorldView2, RapidEye, and Landsat ETM+ sensors using pre-defined filter functions available in ENVI 4.8 (ITT Visual Information Solutions). Next, we resampled the simulated images to the spatial resolution of these sensors, 5 m for RapidEye and 30 m for Landsat ETM+, using pixel aggregate and bilinear convolution methods when appropriate in ENVI 4.8. We pan-sharpened the 30 m simulated Landsat imagery to 15 m spatial resolution using the Gram-Schmidt pan-sharpening method available in ENVI 4.8 [77]. Since the spatial resolution of the original AVIRIS imagery was 3.5 m and it is technically impossible to get better resolution imagery by resampling a coarser resolution image, we did not recreate the 2 m WorldView2 resolution for the simulated WorldView2 imagery. We will refer to these resampled image data in our study as AVIRIS_WV2_, AVIRIS_RE_, AVIRIS_LS-30m_ and AVIRIS_LS-15m_.

#### 2.3.2. Multispectral Sensor Data

**Figure 3:**
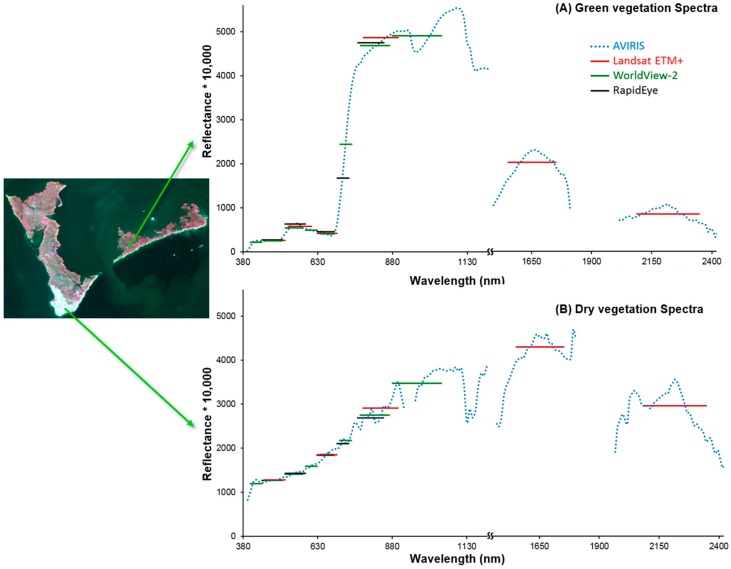
An example of (**A**) green vegetation and (**B**) non-photosynthetic vegetation (NPV) pixel spectra from AVIRIS, WorldView2, RapidEye, and Landsat ETM+ showing the range and spectral resolution of the sensors.

In addition to varying spatial and spectral resolution, sensors also differ in other characteristics, such as lens distortion and signal-to-noise parameters, which can affect their performance in measuring the phenomena of interest [64,66]. Hence, the next step in our study was to compare the ability to detect oil-induced vegetation stress from images acquired by WorldView2, RapidEye, and Landsat 7 ETM+ sensors to that of the AVIRIS image using dates as close to the AVIRIS image date as was available. The WorldView2 and Landsat images were acquired within a week of the AVIRIS image acquisition date while the RapidEye image was within a month of that date (Table 1). The Landsat ETM+ image was further pan-sharpened to 15 m pixel resolution using the Gram-Schmidt pan-sharpening method available in ENVI 4.8 [77]. Figure 3 illustrates the different spectral resolutions of the four sensors.

#### 2.3.3. Selection of Oiled and Oil-Free Areas

We followed a consistent procedure to assign shorelines as either oiled or oil-free and extract pixels along those shorelines for analysis. First, based on the classification of the 2010 AVIRIS image dataset, we used the boundary of the land and water classes to produce a vector layer of the shoreline. Next, sections of the shore with oiled pixels adjacent to them were considered as oiled shoreline. Sections of shore next to oil-free pixels were considered as oil-free shoreline. Only oil-free shoreline within 140 m of an oiled shoreline was used for comparison to ensure that the sites used were as similar to each other as possible, except in the degree of oiling. Finally, we extracted all pixels within 60 m perpendicular to and inland from shore of the selected shorelines compare vegetation stress between oiled and oil-free shorelines.

We subdivided the extracted pixels from the images into zones parallel to the shoreline. A zone is defined as the line of pixels parallel to the shore where each pixel within that zone is away from the shore by the same distance measured in pixel width. For example, zone 1 corresponds to the first pixel adjacent to the shore, zone 2 corresponds to two pixels from the shore, and so on. The width of the zones was determined by the image pixel size. We chose to compare corresponding zones parallel to the shoreline because wetland species distributions, abundance and changes in composition follow elevation gradients [78]. In the gulf wetlands the remote sensing data reveals an inherent spatial pattern irrespective of other impacts: vegetation is moderately dense in the intertidal zone followed by a band of more dense and green vegetation that occurs just beyond the intertidal zone, and then slightly lower density in the inner marsh [20]. Thus by comparing within zones, we minimized the confounding effects of these inherent spatial patterns in wetland vegetation communities.

#### 2.3.4. Detection of Vegetation Stress

We calculated four vegetation indices based on the Green, Red, NIR and SWIR bands: Normalized Difference Vegetation Index (NDVI), Normalized Difference Infrared Index (NDII), Angle at Near InfraRed (ANIR) and Angle at Red (ARed; Table 2). The bandwidth and wavelength centers of the bands in these general electromagnetic regions were dependent on the sensor specifications, e.g., while AVIRIS had narrow 10–15 nm bands, the three multi-spectral sensors had much broader bands from 40 nm to 260 nm (Table 1). For WorldView2, RapidEye, AVIRIS_WV2_ and AVIRIS_RE_, we only calculated NDVI and ARed since both ANIR and NDII require a SWIR band, which WorldView2 and RapidEye lack. For the Landsat and AVIRIS_LS_ images, we calculated all four indices. We did not radiometrically calibrate the different sensor images because our comparisons are between pixels of the same image rather than between sensors. The purpose is to compare the ability of sensors to differentiate healthy vegetation from stressed vegetation within the same image and this can be done effectively by using a statistical approach that is independent of the range and magnitude of index values and then comparing the statistic across sensors.

Effect size is a statistic that measures the degree of overlap between the frequency distributions of two samples; if the frequency distributions overlap it suggests the two samples come from the same population i.e., are not differentiable, while when there is little or no overlap, it suggests that the two samples are not from the same population. The greater the overlap in the frequency distributions, the lower the effect size and vice versa. Effect size is a good metric because it is independent of sample size and the range of index values. To determine differences in plant stress along oiled vs. oil-free shorelines, we first tested whether average index values were different in pixels alongside oiled and oil-free shorelines by using a *t*-test [81]. To assess the magnitude of the differences in index values, we calculated the effect size, Cohen’s d [82]. Cohen’s d is a standardized metric of the difference between two means as it divides the population mean by the pooled standard deviation, allowing for comparisons across many variables and sensors [82,83]. A Cohen’s d value of 0.8 indicates a strong effect, i.e., little overlap between the two frequency distributions suggesting a strong ability to differentiate between the two samples, a value of 0.5 indicates a moderate effect, while a value lower than 0.2 indicates a weak effect [82].

## 3. Results

### 3.1. AVIRIS Hyperspectral Data

The first 14 m (4 pixels) along the oiled shore in the AVIRIS hyperspectral imagery have significantly more stressed vegetation than the corresponding oil-free zone. All indices had significantly lower values (ANIR has the reverse order with higher values indicating lower stress) along the oiled shoreline relative to the oil-free shore line, (Table 3). Despite all zones showing significant differences in index values, the effect size decreased as we moved inland from the shore indicating that oil-induced vegetation stress decreased away from the shore (Table 3).

While all four indices detect plant stress effectively, ARed (average Cohen’s d = 0.81) and NDII (average Cohen’s d = 0.80) exhibited the strongest effect sizes, thus performing better at differentiating vegetation stress due to oil contamination. In the first zone, where the oil impact was expected to be maximum, average value of ARed dropped from 5.12 along oil-free shores to 4.11 along oiled shorelines, while average value of NDII dropped from 0.51 to 0.33 (Table 3). Hence, we selected ARed for a comparison of all four sensors and NDII for an additional comparison of Landsat ETM+ and AVIRIS.

#### 3.1.1. Spectral Resolution

Comparing the ability to detect oil-induced stress in vegetation using AVIRIS_WV2_ vs. AVIRIS data shows the effect of spectral resolution on oil impact detection. We found that effect size for ARed decreased about 12% from AVIRIS narrow-band imagery (average for zones 1–3, Cohen’s d = 0.81) to AVIRIS_WV2_ broad-band imagery (average for zones 1–3, Cohen’s d = 0.71) (Table 4). In the fourth zone from the shore, ARed values between oiled and oil-free shorelines were no longer significantly different using AVIRIS_WV2_ data while they were still significant using AVIRIS data (Table 4). Thus, the reduced spectral resolution of the AVIRIS_WV2_ resulted in a lower ability to discriminate vegetation stress in the most oiled zones, and a lack of differentiation potential in the zone, 14 m inland from shoreline (Table 4). 

#### 3.1.2. Spatial Resolution

**Figure 4:**
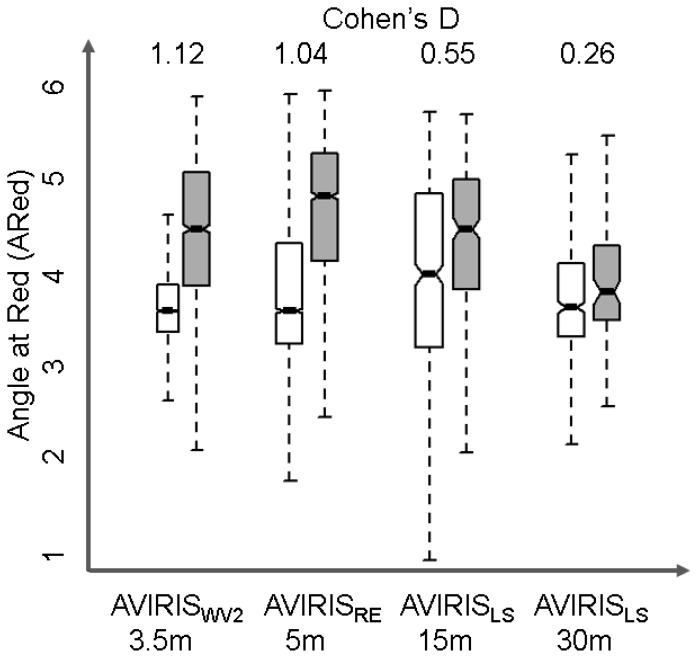
Boxplots of the Angle at Red index in the first pixel zone along oiled (white) vs. oil-free (gray) shoreline using AVIRIS imagery resampled to WorldView2, Landsat ETM+ 15 m and Landsat ETM+ 30 m. Notches on the boxplots indicate the 95% confidence interval. Cohen’s d values are shown for reference at the top.

AVIRIS, AVIRIS_RE_, AVIRIS_LS-15m_ and AVIRIS_LS-30m_ represent progressively coarser spatial resolutions from 3.5 m to 5 m to 15 m to 30 m pixels. Comparing the performance of the four image datasets showed that loss of spatial resolution resulted in especially low effect sizes, i.e., decreased ability to detect differences in vegetation index values. A comparison of the first zone next to the shore shows effect sizes dropped one order of magnitude, from 1.12 to 0.19, as spatial resolution became coarser from 3.5 m AVIRIS_WV2_ to 5 m AVIRIS_RE_ to 15 m AVIRIS_LS_ to 30 m AVIRIS_LS_ (Figure 4; Table 4). For example, the effect size for AVIRIS_LS_ dropped by half, from 0.55 in the 15 m pixel image to 0.26 in the 30 m pixel image, indicating that the area of overlap between the oiled and oil-free index frequency distributions increased considerably.

**Figure 5:**
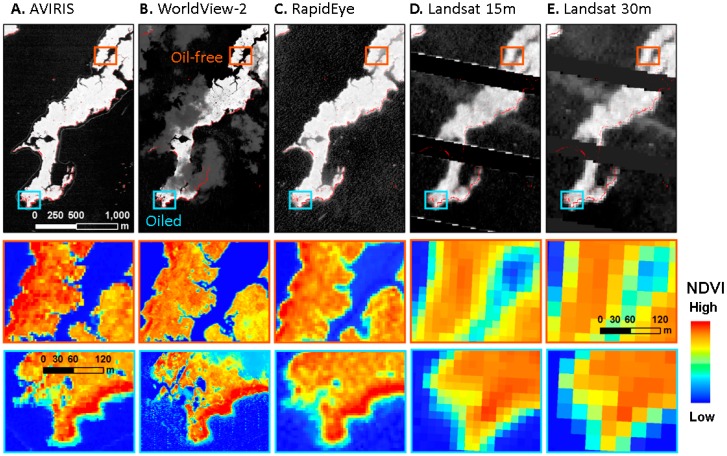
A subset of Barataria Bay shown in gray scale (**A**) AVIRIS, (**B**) WorldView-2, (**C**) RapidEye, (**D**) Landsat ETM+ pan sharpened 15 m, (**E**) Landsat ETM+ 30 m with oiled pixels overlaid in red. NDVI color map of an oiled and an oil-free area is detailed in the second and third rows from the top. Note the lack of spatial definition of the affected area as spatial resolution deteriorates.

While all four spatial resolutions were sufficient to detect significant vegetation stress in the pixels located largely within the 14 m zones next to the oiled shoreline, the sensitivity of that detection reduced quickly with increasing pixel size. The zone of oil impact became less defined as zones became more mixed (Figure 5).

### 3.2. Multispectral Data

**Figure 6:**
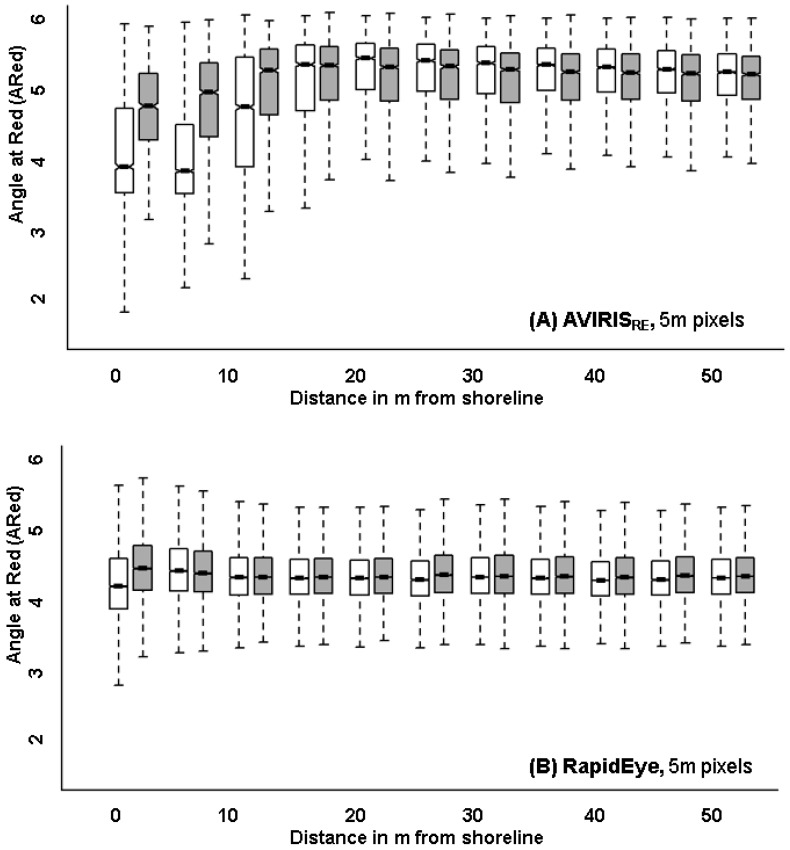
Angle at Red vs. distance from shoreline in meters for (**A**) AVIRIS resampled to RapidEye and (**B**) actual RapidEye imagery. Distribution of the index along the oiled shoreline is in white and along oil-free shoreline, in gray.

**Figure 7:**
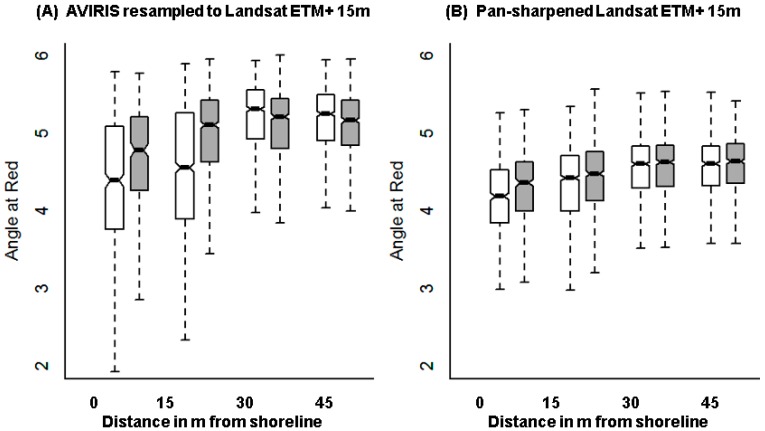
Angle at Red vs. distance from shoreline in meters for (**A**) AVIRIS resampled to Landsat 15 m and (**B**) actual Landsat ETM+ 15 m pan-sharpened imagery. Distribution of the index along the oiled shoreline is in white and along oil-free shoreline is in gray.

All three multispectral sensors, WorldView2, RapidEye and Landsat ETM+, performed worse than the AVIRIS imagery resampled to the spatial and spectral resolution of these sensors (Table 5). Using original WorldView2 imagery, we were able to detect significant differences in index values along oiled vs. oil-free shorelines in zones up to 10 m inland (5 pixels; Table 5), which is similar to the 10.5 m distance over which significant differences were observed for AVIRIS_WV2_. However, the effect sizes for WorldView2 data were lower than those for AVIRIS_WV2_ data indicating weaker differentiation in vegetation index values. The RapidEye imagery could only detect impact 5 m inland (1 pixel), while AVIRIS_RE_ detected oil impact up to 15 m (3 pixels). Again, the RapidEye effect size was less than half that of AVIRIS_RE_ for the corresponding zone (Figure 6, Table 5). Similarly, for Landsat, effect sizes dropped to half of those using the resampled AVIRIS_LS-15m_ imagery (Figure 7).

Landsat comparisons using NDII also showed that AVIRIS_LS-15m_ imagery produced higher effect sizes than the pan-sharpened Landsat ETM+ 15 m imagery (Table 6). For the Landsat 30 m image, we found no significant differences among oiled and oil-free shores while the AVIRIS_LS_-_30m_ was still able to differentiate oiled conditions for the first 30 m inland (1 pixel).

## 4. Discussion

Selecting the appropriate remote sensing data to detect and monitor impacts of oil spills and recovery involves a careful consideration of cost and ability to achieve goals as no single sensor meets all the requirements needed for achieving such a task. Here, we set out to assess whether sensor spatial and spectral resolution, and sensor characteristics influence the ability to detect and map vegetation stress and mortality due to oil spills. 

However, before we can assess vegetation stress, it is important to have a method to distinguish stress due to oiling from stress due to other causes such as hurricanes, drought, etc. Vegetation responds to completely different stress factors such as drought or oiling, in similar ways by losing pigment and water ultimately leading to plant mortality. Hence, any vegetation stress in the affected area cannot automatically be attributed to oil contamination. There are three potential ways to measure the effect of oiling on vegetation. First, a comparison of pre-spill imagery to post-spill imagery of the oil-spill region can determine loss of plant health due to oiling. However, as oil spills occur without warning, pre-spill imagery is not always available. Furthermore, a difference in plant health, if observed, might still be due to seasonal changes such as the arrival of the dry season. Second, an oil susceptibility analysis might help identify areas with higher probability of oiling and possibly even identify degree of vulnerability to oil impacts [84,85]. If the vegetation stress maps coincide with the susceptibility map predictions, it would potentially indicate oil effects. Third, if oil contamination can be mapped in the post-spill imagery, then vegetation in oiled areas can be compared to oil-free areas within the same region. Assuming that other sources of stress will affect the entire region, any differential in vegetation stress between oiled and oil-free areas could reasonably be attributed to oil contamination [20]. Mapping oil on land is harder than mapping it on water. Many studies have mapped oil spills on water with multispectral imagery [30,86,87], hyperspectral imagery [88,89], and RADAR [90,91,92]. But mapping oil on land requires spectroscopy data acquired at high spatial resolution [20]. Since the DWH oil spill was the largest oil spill in US history, the gulf shoreline was extensively mapped and surveyed with the help of airborne hyperspectral data to determine extent of oiling [20,62,63,93]. Hence this study uses the third approach to determine the impact of oil on wetland health.

### 4.1. Spectral Resolution

AVIRIS_WV2_ has the same spatial resolution as AVIRIS in this study (3.5 m) but broad-bands of 40–180 nm width instead of the 10 nm narrow-bands of the AVIRIS sensor. Thus, the effect spectral resolution is examined by comparing their performance. Several studies have shown that fine spectral resolution is important for species-level classification [22,94,95,96], in some cases, even more important than spatial resolution [97,98]. However, when the goal is to detect plant stress, especially when using indices, coarser spectral resolution may be sufficient, particularly when combined with fine spatial resolution [99,100]. Teillet et al. [99] calculated NDVI at various spatial and spectral resolutions and found that when bandwidth of the Red band increased beyond 50 nm, there was an appreciable drop in the ability of the index to track plant health, and found similar results for other indices. As the Red band width for all three multispectral sensors we used is greater than 50 nm, this is consistent with the above reported decrease in ability to track plant health (Red bandwidth, RapidEye: 55 nm, WorldView2: 60 nm, and Landsat ETM+: 60 nm). Hence, it is expected that the loss of spectral resolution will degrade the ability to discriminate oil-induced plant stress. Our study demonstrated that there was loss of ability to detect vegetation stress with broader spectral bands, as AVIRIS was able to detect significant differences in vegetation index values up to 14 m inland (inland zones with lower oil impact) but AVIRIS_WV2_ could not. Additionally, index value differences were weaker when using AVIRIS_WV2_ in all zones. 

Multiple studies have also pointed out the importance of a wider spectral range, specifically the advantage of having SWIR bands for detecting vegetation and vegetation stress [101,102,103]. Of the three multispectral sensors we tested, only Landsat ETM+ has SWIR bands, which we used to calculate NDII. The results of the Landsat NDII comparison showed that for AVIRIS_LS-15m_, AVIRIS_LS-30m_ and LS-15 m there was a stronger differentiation between oiled and oil-free shorelines using NDII than using ARed (no SWIR band). However, with the LS-30 m sensor data, ARed detected a significant response while NDII did not, indicating that this wider spectral range is not always necessary, which is likely due to an interaction with the effect of spatial resolution. However, such interactions between wider spectral range and spatial resolution need further investigation. Furthermore, a comparison of ARed and NDII using AVIRIS 3.5 m narrow-band data showed that ARed performed as well as NDII, again indicating that the presence of the SWIR band might not be critical to detection of vegetation stress. This could be because vegetation stress is identifiable across multiple regions of the electro-magnetic spectrum [37]. 

### 4.2. Spatial Resolution

The choice of the appropriate spatial resolution is important for the detection of oil-induced vegetation stress, and critical for the precise identification of the boundaries of the affected area. In this particular study, the impact was localized to a narrow 14 m band along the affected shoreline [20], and the degree of impact differed with distance from the shore. Hence it was important to have a spatial resolution finer than 14 m to delineate this oil impact. These impacts were easily detected at all resolutions except at Landsat 30 m, but effect sizes declined rapidly as spatial resolution became coarser. At the Landsat 30 m pixel resolution, half of the area within the first pixel next to the shore is not affected by oil and the impacted vegetation is intermixed with healthy vegetation. This reduces the effect size, making it difficult to detect significant differences between stressed and non-stressed vegetation. However, to detect the gradient of stress inland from the shoreline, and to delineate the affected area with some degree of confidence, a finer spatial resolution is required as demonstrated by our results. Studies by Teillet et al. [99] and Paul et al. [104] suggested that required spatial resolution depends on the study goals and that coarse spatial resolution was sufficient for studies involving large targets. For example, Paul et al. [104] were trying to map the extent of debris-free glaciers wider than 100 m and found the Landsat TM resolution of 30 m to be sufficient. The results of our study corroborate this assertion that the aims of the study determine the resolution required. In this study, Landsat pan-sharpened 15 m imagery would have been sufficient to determine the length of shoreline affected by the oil spill, but would not be able to identify the depth of penetration or delineate the affected area. By using finer spatial resolution sensors like AVIRIS or WorldView2, we were able to examine depth and magnitude of oil impacts on vegetation. Our study site, Barataria Bay, has a consistent topography where a gradual increase in elevation at the shore creates distinct subtidal, intertidal and upland areas. The oil, coming in from offshore, penetrated to the high-tide mark thus confining the impact of the spill in a narrow 14 m band by the shoreline [20]. However, in other regions in the gulf, a different hydrology and topography forced a different outcome from the DWH oil spill [105]. For example, in low-lying Chandeleur Island mangroves, the tide penetrated much further spreading oil more uniformly and likely causing mortality due to coating of pneumatophores rather than the mangrove canopy and leaves [105]. Thus, in the mangrove ecosystem, coarser resolution imagery might have proved sufficient for identifying the impacted coastline as well as the area affected.

### 4.3. Sensor Signal-to-Noise Ratio (SNR)

While all multispectral sensors were successful in detecting significant vegetation stress along the oiled shores relative to oil-free shores, their performance was consistently inferior to AVIRIS. This remained true even when AVIRIS was spectrally and spatially resampled to match these other sensors. This suggests that factors other than spectral and spatial resolution also play a role in the performance. 

The radiometric resolution of the four sensors tested in this study ranged from 8-bit quantization in Landsat images to 16-bit quantization for AVIRIS and RapidEye (Table 1). Since oil darkens the albedo in most bands from the visible to the SWIR, the radiometric *range* is unlikely to have a large impact on performance. Moreover, RapidEye performed no better than WorldView2 despite having higher radiometric *resolution*, suggesting that radiometric resolution did not affect performance. However, RapidEye has coarser spatial resolution compared to WorldView2 hence it is not possible to completely isolate the effect of radiometric resolution from the effect of spatial scale. Therefore, more specific tests might be needed to examine how radiometric resolution affects performance.

The signal-to-noise ratio of AVIRIS in both the visible and NIR regions is better than any of the other sensors [64,65,66,67,68]. Additionally, when AVIRIS was resampled to the multispectral sensors’ characteristics, it likely performed better because each new band was calculated from the sum of several narrow bands with high SNR, increasing the signal strength of the resampled image [64]. Better SNR implies that the sensor is more likely to detect vegetation stress when present, and also more likely to discriminate small differences in vegetation stress. There are very few studies that compare the performance of different sensors based on their SNR. Platt and Goetz [64] compared AVIRIS performance to Landsat and concluded that the advantage of AVIRIS over Landsat when classifying land cover was due to spectral resolution, not SNR. However, the current study suggests that the AVIRIS signal is stronger compared to that of the other sensors, even when spectrally resampled. 

Factors other than SNR may also have influenced the performance of the multispectral sensors. Both WorldView2 and RapidEye images were acquired off-nadir at oblique angles which can increase scattering across pixels, therefore making it more difficult to separate oiled from non-oiled areas. Additionally, multispectral sensors lack the narrow bands that allow accurate estimates of water vapor in the atmosphere and help achieve a better atmospheric calibration. Current (e.g., Landsat 8, WorldView3, Sentinel-2) and future sensors may have SNR comparable to AVIRIS. A comparison with these sensors might yield different results. Landsat 8 was launched in 2013, WorldView3 in 2014, and Sentinel-2 in 2015, hence they were unavailable at the time of this oil spill in 2010, but could be useful for future disasters.

### 4.4. Timing of Image Acquisition

The WorldView2 imagery, while successful in detecting oil impacts, indicated a narrower shoreline zone affected by oil compared to the AVIRIS imagery. This is likely because the WorldView2 data were acquired at high tide. Tide levels were 29 cm higher at the time the WorldView2 images were acquired, compared to the AVIRIS image (NOAA, http://tidesandcurrents.noaa.gov/). This highlights the importance of tidal effects when the study area is dynamic and water levels are changing throughout the day. It is important to schedule image acquisitions (where possible e.g., in the case of airborne imagery) at low tide to expose maximum possible land area. In our study, this was especially important because the oil came inland from the shoreline resulting in the most affected area being next to the shore. The ability to control the timing of image acquisition is also important during the wet season or in high-rainfall regions like the tropics. The WorldView-2 imagery was also the one with the most cloud cover and parts of the oiled shoreline were under clouds and could not be analyzed at all. The difference in timing between the AVIRIS and RapidEye imagery also likely explains the poor performance of Rapid Eye, which was collected almost a month later than the data from the other sensors. With satellite imagery, there is very little control over the time of acquisition which can result in underestimation of the affected area and its impacts on vegetation stress.

## 5. Conclusions

Multispectral sensor imagery is relatively more economical than hyperspectral imagery and is now often available at a fine spatial resolution making it an excellent tool for mapping impact and monitoring recovery of ecosystems after environmental disasters such as oil spills, floods, and hurricanes. This study examined the potential of three multispectral sensors with considerably less financial costs than AVIRIS (i.e., WorldView2 is estimated at half the cost of AVIRIS, RapidEye at a fraction of the cost, and Landsat ETM+ is free) for mapping the extent of the impact of the 2010 DWH oil spill on the wetlands of Louisiana. If accuracy is prioritized, clearly AVIRIS data performed the best, even when resampled to coarser spectral and spatial resolutions. This indicates that sensor characteristics, such as signal-to-noise ratio, are critical in detecting vegetation stress. WorldView2 and RapidEye were unable to detect oil impact to the same inland distance as AVIRIS. In the case of WorldView2 this might be because of high tides covering oiled areas denuded of vegetation, and in the case of RapidEye the late acquisition date. Landsat ETM+ 15 m data gave mixed results likely due to its coarse spatial resolution. 

Spatial resolution was therefore the most critical factor limiting the detection of the extent of oil impact although spectral resolution and sensor characteristics (e.g., SNR, sensor view angle, etc.) also limited the ability to detect vegetation stress. Based on these results we suggest that since no sensor has all of the characteristics necessary to detect extent, impact, and recovery from oil spills, it is important to determine which sensor is best for the objectives of a particular project. If the objective is to map the extent of oil impact, sensors like WorldView2 and RapidEye, or even Landsat, are sufficient if the impact is widespread. If the objective is to detect vegetation stress and recovery, and delineate the affected area for treatment, then it is important to have high spectral and spatial resolution collected at the optimal time to reveal the stress, as these characteristics allow for detecting even subtle differences in vegetation condition. The results of this study provide important guidelines for sensor selection which requires careful consideration of the extent of impact, location of the affected area, degree of complexity required in detection of impact, and characteristics of the study area which might require specific timing of image acquisition. In mapping the impact of an event such as an oil spill, additional fine resolution imagery might be required to map the extent of oil after the spill since multispectral imagers don’t have the necessary bands to detect oil presence on soil or vegetation.

## Figures and Tables

**Table 1:** Characteristics of the four sensors and date of image acquisition for data analyzed in this study.

	AVIRIS	WorldView2	Rapid Eye	Landsat ETM+
Bandwidth	10–15 nm	40–180 nm	40–90 nm	60–260 nm
Spatial resolution	3.5 m	2 m	5 m	15 m, 30 m
Radiometric resolution	16-bit	11-bit	16-bit	8-bit
Time of acquisition	19 September 2010	8 September 2010	8 October 2010	13 September 2010
Signal-to-noise ratio	800–1200 [64]	250–500 [65]	90–140 [66,67]	20–55 [64,68]
Cloud cover	0%	30%	0%	0%

**Table 2:** Vegetation indices used to test for the effects of oil contamination on vegetation stress, and the sensors they were calculated for. R_G_, R_R_, R_NIR_ and R_SWIR_ are the reflectance values in the Green (G), Red (R), Near InfraRed (NIR) and ShortWave InfraRed (SWIR) bands respectively and λ_G_ (550–570 nm), λ_R_ (650 nm), λ_NIR_ (805–840 nm) and λ_SWIR_ (1650 nm) are the wavelength values at the band centers.

Inputs	Formula	Relevance	References	Index Calculated Using Sensors
Normalized Difference Vegetation Index (NDVI)	$\frac{R_{NIR}-R_{R}}{R_{NIR}+R_{R}}$	Index of green plant cover and LAI	[46,47]	AVIRIS
Normalized Difference Infrared Index (NDII)	$\frac{R_{NIR}-R_{SWIR}}{R_{NIR}+R_{SWIR}}$	Sensitive to plant water content	[43,79]	AVIRIS, Landsat
Angle at NIR (ANIR) (rad)	Angle between (R_R_, λ_R_), (R_NIR_, λ_NIR_), and (R_SWIR_, λ_SWIR_)	Angle index sensitive to change in land cover type	[44,80]	AVIRIS
Angle at Red (ARed) (rad)	Angle between (R_G_, λ_G_), (R_R_, λ_R_), and (R_NIR_, λ_NIR_)	Angle index sensitive to plant pigments and land cover type	[20,80]	AVIRIS, WorldView2, RapidEye, Landsat

**Table 3:** Student *t*-test comparison results for four indices calculated with AVIRIS data. Value of Cohen’s d indicates effect size.

Index	Zone	N		Mean		Std. Dev.		Student *t*-Statistic	*p*-Value	Cohen’s d
		Oiled	Oil-Free	Oiled	Oil-Free	Oiled	Oil-Free			
NDVI	1	5539	3156	0.474	0.583	0.223	0.227	−21.560	0.000	0.483
	2	5220	3118	0.618	0.676	0.178	0.192	−13.614	0.000	0.314
	3	3941	2440	0.683	0.711	0.159	0.153	−6.933	0.000	0.177
ARed	1	5539	3156	4.113	5.118	0.796	0.760	−58.320	0.000	1.284
	2	5220	3118	4.596	5.287	0.944	0.752	−36.862	0.000	0.789
	3	3941	2440	5.122	5.397	0.834	0.665	−14.585	0.000	0.357
	4	3841	2533	5.399	5.449	0.670	0.593	−3.088	0.002	0.077
NDII	1	5539	3156	0.333	0.510	0.172	0.155	−49.431	0.000	1.072
	2	5220	3118	0.395	0.531	0.172	0.132	−40.495	0.000	0.858
	3	3941	2440	0.484	0.548	0.151	0.121	−18.691	0.000	0.457
	4	3841	2533	0.539	0.561	0.125	0.113	−7.118	0.000	0.179
ANIR	1	5539	3156	1.531	0.775	0.849	0.708	44.471	0.000	0.944
	2	5220	3118	0.940	0.503	0.802	0.554	29.361	0.000	0.608
	3	3941	2440	0.584	0.424	0.601	0.425	12.500	0.000	0.298
	4	3841	2533	0.461	0.422	0.484	0.411	3.376	0.001	0.084

**Table 4:** Student *t*-test comparison results for ARed in radians calculated from AVIRIS narrow-band image and AVIRIS imagery spectrally and spatially resampling to WorldView2 (AVIRIS_WV2_), RapidEye (AVIRIS_RE_), and Landsat (AVIRIS_LS_) 15 m and 30 m. Value of Cohen’s d indicates effect size.

Sensor	Zone	N		Means		Std. Dev.		Student *t*-Statistic	*p*-Value	Cohen’s d
		Oiled	Oil-Free	Oiled	Oil-Free	Oiled	Oil-Free			
AVIRIS 3.5 m	1	5539	3156	4.113	5.118	0.796	0.760	−58.320	0.000	1.284
	2	5220	3118	4.596	5.287	0.944	0.752	−36.862	0.000	0.789
	3	3941	2440	5.122	5.397	0.834	0.665	−14.585	0.000	0.357
	4	3841	2533	5.399	5.449	0.670	0.593	−3.088	0.002	0.077
AVIRIS_WV2_ 3.5 m	1	5539	3156	3.861	4.620	0.657	0.712	−49.130	0.000	1.120
	2	5220	3118	4.238	4.787	0.805	0.760	−31.257	0.000	0.697
	3	3941	2440	4.681	4.927	0.792	0.714	−12.853	0.000	0.323
AVIRIS_RE_ 5 m	1	3965	2271	0.965	1.696	0.724	0.660	−40.632	0.000	1.043
	2	3785	2222	1.538	1.902	0.805	0.673	−18.816	0.000	0.480
	3	2759	1887	1.949	1.989	0.684	0.634	−2.043	0.041	0.060
AVIRIS_LS_ 15 m	1	1318	852	4.557	4.953	0.776	0.631	−13.040	0.000	0.549
AVIRIS_LS_ 30 m	1	749	405	4.921	5.071	0.625	0.442	−4.723	0.000	0.264

**Table 5:** Student *t*-test comparison results for ARed in radians calculated from AVIRIS imagery spectrally and spatially resampled to WorldView2 (AVIRIS_WV2_), RapidEye (AVIRIS_RE_), Landsat (AVIRIS_LS_) 15 m and 30 m compared to the actual sensor imagery. Value of Cohen’s d indicates effect size.

Sensor	Zone	N		Means		Std. Dev.		Student *t*-Statistic	*p*-Value	Cohen’s d
		Oiled	Oil-Free	Oiled	Oil-Free	Oiled	Oil-Free			
AVIRIS_WV2_	1	5539	3156	3.861	4.620	0.657	0.712	−49.130	0.000	1.120
3.5 m	2	5220	3118	4.238	4.787	0.805	0.760	−31.257	0.000	0.697
	3	3941	2440	4.681	4.927	0.792	0.714	−12.853	0.000	0.323
	1	5111	2223	3.553	3.721	0.594	0.606	−11.017	0.000	0.282
	2	6506	3067	3.577	3.938	0.462	0.531	−32.310	0.000	0.744
WV2 2 m	3	6449	3202	3.794	4.013	0.546	0.549	−18.549	0.000	0.402
	4	4992	2576	3.986	4.093	0.575	0.591	−7.519	0.000	0.184
	5	4839	2588	4.114	4.161	0.544	0.557	−3.493	0.000	0.086
	1	3965	2271	0.965	1.696	0.724	0.660	−40.632	0.000	1.043
AVIRIS_RE_ 5 m	2	3785	2222	1.538	1.902	0.805	0.673	−18.816	0.000	0.480
	3	2759	1887	1.949	1.989	0.684	0.634	−2.043	0.041	0.060
RE 5 m	1	14979	10495	4.238	4.456	0.496	0.449	−36.615	0.000	0.458
AVIRIS_LS_ 15 m	1	1318	852	4.557	4.953	0.776	0.631	−13.040	0.000	0.549
Landsat 15 m	1	596	392	4.177	4.281	0.484	0.477	−3.330	0.001	0.216
AVIRIS_LS_ 30 m	1	749	405	4.921	5.071	0.625	0.442	−4.723	0.000	0.264
Landsat 30 m	1	467	98	3.725	3.885	0.685	0.564	−2.453	0.015	0.240

**Table 6:** Student *t*-test comparison results for NDII calculated from AVIRIS imagery spectrally and spatially resampled to Landsat (AVIRIS_LS_) 15 m and 30 m compared to the actual Landsat imagery. Value of Cohen’s d indicates effect size.

Sensor	Zone	N		Means		Std. Dev.		Student *t*-Statistic	*p*-Value	Cohen’s d
		Oiled	Oil-Free	Oiled	Oil-Free	Oiled	Oil-Free			
AVIRIS_LS_ 15 m	1	1318	852	0.431	0.540	0.172	0.130	16.772	0.000	0.695
Landsat 15 m	1	596	392	0.221	0.252	0.060	0.047	−9.279	0.000	0.573
AVIRIS_LS_ 30 m	1	749	405	0.498	0.553	0.120	0.100	−8.359	0.000	0.489
Landsat 30 m	1	467	98	0.254	0.397	0.422	0.907	−1.517	0.132	*

* Cohen’s d not reported for Landsat ETM+ 30 m image because the *p*-value is not significant.
